# Ifn1 is an intracellular GMP 5′-nucleotidase induced during the fission yeast response to phosphate starvation

**DOI:** 10.1128/mbio.03942-25

**Published:** 2026-02-26

**Authors:** Aleksei Innokentev, Beate Schwer, Stewart Shuman

**Affiliations:** 1 Molecular Biology Program, Memorial Sloan Kettering Cancer Center 5803, New York, New York, USA; 2 Department of Microbiology and Immunology, Weill Cornell Medical College 12295 , New York, New York, USA; Instituto Carlos Chagas, Curitiba, Brazil

**Keywords:** phosphate homeostasis, 5′-nucleotidase, aspartyl-phosphatase, NMP specificity

## Abstract

*Schizosaccharomyces pombe* adapts to phosphate starvation by upregulating the expression of (i) a cell-surface acid phosphatase, Pho1, that mobilizes inorganic phosphate from the extracellular milieu; (ii) transmembrane transporters that take up inorganic phosphate (Pho84, Pho841, and Pho842) and glycerophosphocholine (Tgp1); and (iii) secreted extracellular 5′-nucleotidase enzymes (Efn1 and Efn2) that release inorganic phosphate from rNMPs, with a preference for CMP. The expression of SPAC24B11.05, a fission yeast homolog of the budding yeast 5′-nucleotidase Sdt1, is upregulated during phosphate starvation, and the protein accumulates without being secreted. Here, we characterized recombinant SPAC24B11.05 (herein Ifn1, for intracellular 5′-nucleotidase) as a Mg^2+^-dependent phosphohydrolase of the aspartyl-phosphatase (HAD) superfamily. Unlike Sdt1, which is specific for pyrimidine mononucleotides and nicotinamide mononucleotide (NMN), Ifn1 displays a preference for hydrolysis of GMP > IMP > CMP > AMP > UMP and is unable to hydrolyze NMN. Ifn1 activity is abolished by alanine mutations of the Asp11 nucleophile of the signature ^11^**D**LDNC^15^ motif and by alanines in lieu of Asp80 and Asp174 that are predicted to coordinate the ribose hydroxyls and the metal cofactor, respectively. Changing Ifn1 Arg50, which is predicted to engage the guanine nucleobase, to Asn, the corresponding residue in Sdt1, enhances hydrolysis of CMP and AMP and suppresses hydrolysis of GMP, IMP, and UMP, with no gain of activity with NMN. We find that overexpression of catalytically active Ifn1 is toxic to fission yeast.

**IMPORTANCE:**

Phosphate starvation in fission yeast triggers increased expression of enzymes with imputed roles in phosphate dynamics. Many starvation-induced phosphohydrolases are annotated as acting on nucleotides, though their substrate specificities have not been interrogated. Here, we characterize fission yeast Ifn1 as a starvation-induced 5′-nucleotidase of the aspartyl-phosphatase (HAD) superfamily with a preference for hydrolysis of GMP and IMP that distinguishes it from the homologous budding yeast pyrimidine-specific 5′-nucleotidase Sdt1. A single swap of Ifn1 Arg50 to Asn (the equivalent position in Sdt1) elicits a substrate switch, manifested as a gain of activity with CMP and suppression of activity with GMP and IMP. An emergent theme is that 5′-nucleotidase substrate specificity is a tunable property.

## INTRODUCTION

Fission yeast responds to acute phosphate starvation by inducing the transcription of phosphate acquisition genes encoding secreted or cell-surface-associated enzymes that mobilize phosphate from the extracellular environment and transmembrane transporters of inorganic phosphate or simple phosphate-containing compounds (1–3). Pho1, a histidine acid phosphatase enzyme that hydrolyzes phosphomonoesters, is induced and then accumulates on the cell surface of phosphate-starved cells and in the culture medium (2, 4). Efn1 and Efn2 are paralogous extracellular 5′-nucleotidases of the binuclear metallophosphoesterase enzyme family that are highly induced and secreted during phosphate starvation (2, 4). Efn1 and Efn2 release inorganic phosphate from rNMPs, with a preference for CMP (4). Secretion of Efn1/2 enables phosphate-starved fission yeast to thrive by using extracellular CMP as a source of inorganic phosphate (4). The inorganic phosphate liberated by Pho1 and Efn1/2 is taken up by plasma membrane phosphate transporters Pho84, Pho841, and Pho842 that are also induced during acute phosphate starvation (2). Tgp1, induced during phosphate starvation (1, 2), is a transmembrane transporter of the lipid metabolite glycerophosphocholine (GPC) (5, 6). GPC may serve as a source of phosphate after catabolism by the intracellular enzyme Gde1, which is induced during phosphate starvation and hydrolyzes GPC to choline and glycerol-3-phosphate (2, 6).

Phosphate starvation drives manifold changes in the expression of other intracellular enzymes with known or imputed roles in phosphate dynamics, along with major changes in the intracellular levels of phosphate-containing metabolites (2, 7). For example, the vacuolar storage pool of inorganic polyphosphate is quickly depleted (2), presumably to buffer the immediate effects of acute starvation, as is the intracellular pool of inositol pyrophosphate 1,5-IP_8_ (8) that acts as an agonist of inorganic polyphosphate synthesis in fission yeast (9, 10). A time-resolved metabolome survey of phosphate-starved fission yeast revealed progressive depletion of rNTPs and dNTPs (7). Transcriptome profiling highlighted a variety of upregulated genes encoding intracellular enzymes that are known or are imputed to hydrolyze phosphometabolites (2). Upregulated enzymes with known substrate specificities include the inositol pyrophosphatases Siw14 and Asp1 (11, 12). Multiple other starvation-induced phosphohydrolases are annotated in Pombase as acting on nucleotide substrates (2).

**Fig 1:**
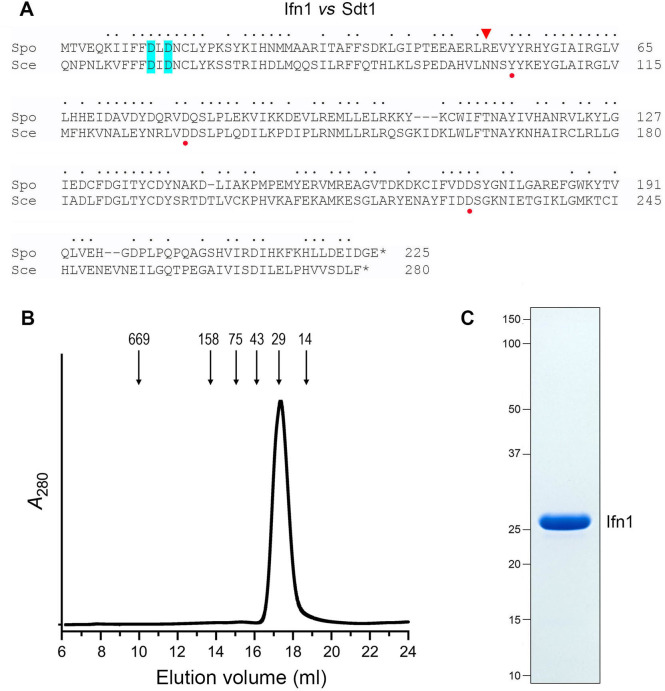
Fission yeast Ifn1. (**A**) Alignment of the amino acid sequences of *S. pombe* Ifn1 (Spo) and *S. cerevisiae* Sdt1. Positions of side chain identity/similarity are indicated by • above the Ifn1 sequence. The two aspartates in the signature DxD active site motif are highlighted in cyan shading. Other conserved active site constituents that were mutated to alanine in the present study are denoted by red dots below the Sdt1 sequence. Ifn1 Arg50, which is replaced by Asn in Sdt1, is indicated by an arrowhead above the Ifn1 sequence. (**B**) Gel filtration. Recombinant tag-free wild-type Ifn1 (0.5 mL, 0.5 mg/mL) was gel filtered through a 24 mL Superdex-200 column. The elution profile was monitored continuously by *A*_280_ as a function of elution volume. Arrows denote the elution peaks and native sizes for a mixture of calibration standards: thyroglobulin (669 kDa), ferritin (440 kDa), aldolase (158 kDa), conalbumin (75 kDa), ovalbumin (44 kDa), carbonic anhydrase (29 kDa), and RNase A (14 kDa). (**C**) An aliquot (10 µg) of the peak Superdex-200 Ifn1 fraction was analyzed by SDS-PAGE. The Coomassie blue-stained gel is shown, with the positions and sizes (kDa) of marker polypeptides indicated on the left.

In the present study, we focus on SPAC24B11.05, a 226-aa protein encoded by the *SPAC24B11.05* gene on chromosome I. The *SPAC24B11.05* mRNA was upregulated by 5- to 10-fold after 4, 8, and 12 h of phosphate starvation, and the cellular level of SPAC24B11.05 protein increased by 4-fold at 8 and 12 h post-starvation (2). No secreted SPAC24B11.05 protein was detected in the culture medium after 12 h of phosphate starvation (4). SPAC24B11.05 is annotated in Pombase as a predicted pyrimidine 5′-nucleotidase based on its homology to the 280-aa *Saccharomyces cerevisiae* pyrimidine 5′-nucleotidase Sdt1 (13, 14). Sdt1 belongs to the haloacid dehalogenase (HAD) superfamily of divalent cation-dependent enzymes that employ a two-step mechanism of phosphate cleavage via a covalent enzyme–(aspartyl-Oδ)-phosphate intermediate (14). Sdt1 is >100-fold more active in hydrolyzing UMP and CMP compared to AMP and GMP (13). Sdt1 also catalyzes the hydrolysis of nicotinamide mononucleotide (NMN), an intermediate in NAD^+^ metabolism (15). A primary structure alignment of SPAC24B11.05 and Sdt1 highlights 132 positions of sidechain identity/similarity, including the signature catalytic DxD motif, in which the first aspartate is the nucleophile for covalent phosphoryl transfer (Fig. 1A). The N-terminal 50-aa segment of Sdt1 has no counterpart in SPAC24B11.05.

Our aims here were to determine whether SPAC24B11.05 is indeed a 5′-nucleotidase and, if so, to elucidate its substrate specificity. We report that recombinant SPAC24B11.05 catalyzes magnesium-dependent hydrolysis of 5′-rNMPs with a substrate preference for GMP that distinguishes it from Sdt1. Moreover, SPAC24B11.05 is unable to hydrolyze NMN. Herein, we refer to SPAC24B11.05 as intracellular five-prime nucleotidase 1 (Ifn1) and its gene as *ifn1*^+^.

## RESULTS

### Recombinant Ifn1 is a divalent cation-dependent 5′-nucleotidase

We produced Ifn1 in *Escherichia coli* as a His_10_Smt3 fusion and purified it from a soluble extract by sequential Ni-affinity chromatography/imidazole elution, removal of the His_10_Smt3 tag by treatment with Ulp1 protease, recovery of the tag-free Ifn1 protein in the flow-through of a second Ni-affinity column, and a final Superdex-200 gel filtration step during which Ifn1 eluted as a single peak, comprising the pure 24 kDa Ifn1 polypeptide (Fig. 1B and C). The elution volume (17.3 mL) relative to calibration standards indicated that Ifn1 is a monomer. To assay 5′-nucleotidase activity, Ifn1 was incubated with 1 mM rNMP substrate at 37°C in the presence of MgCl_2_, and the release of inorganic phosphate was measured colorimetrically using the Malachite Green reagent. Because pilot experiments indicated that GMP was the preferred substrate, we optimized the reaction parameters using 1 mM GMP, as described below.

**Fig 2:**
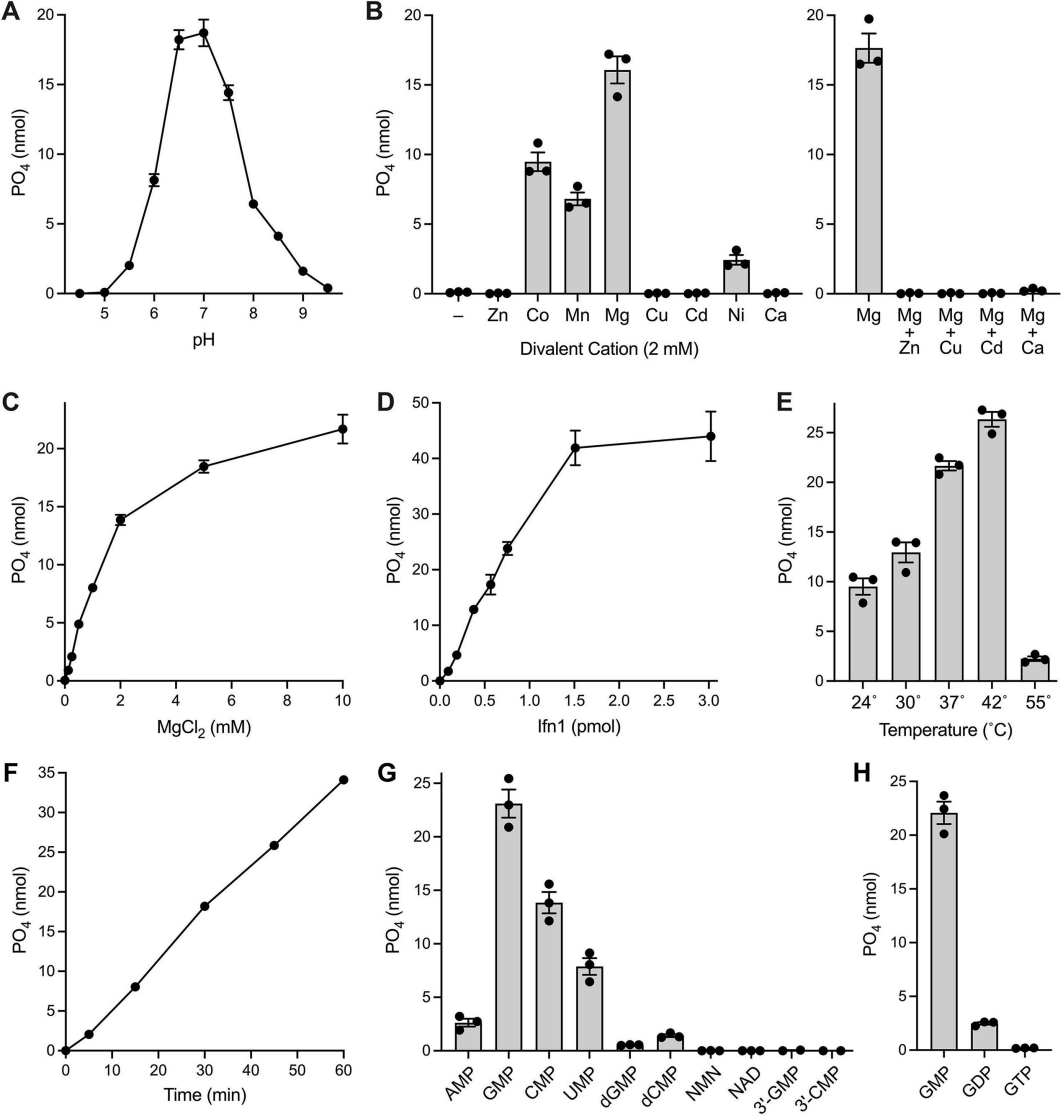
Characterization of the Ifn1 5′ nucleotidase. (**A**) pH profile. Reaction mixtures (50 µL) containing 50 mM Tris buffer (either Tris-acetate pH 4.5, 5.0, 5.5, and 6.0 or Tris-HCl pH 6.5, 7.0, 7.5, 8.0, 8.5, 9.0, and 9.5), 2 mM MgCl_2_, 1 mM GMP, 0.1% (wt/vol) BSA, and 15 ng Ifn1 were incubated for 30 min at 37°C. The extent of phosphate release from GMP is plotted as a function of pH. Each datum is the average of three independent experiments ± SEM. (**B**) Divalent cation specificity. Reaction mixtures (50 µL) containing 50 mM Tris-HCl, pH 7.0, 1 mM GMP, either no added divalent cation (–) or 2 mM divalent cation as specified (added as the chloride salt), 0.1% (wt/vol) BSA, and 15 ng Ifn1 were incubated for 30 min at 37°C. Phosphate release is plotted in bar graph format for each condition tested. Bar height is the average of three independent experiments ± SEM, with individual replicates indicated by filled circles. (**C**) Magnesium titration. Reaction mixtures (50 µL) containing 50 mM Tris-HCl, pH 7.0, 1 mM GMP, MgCl_2_ as specified, 0.1% (wt/vol) BSA, and 15 ng Ifn1 were incubated for 30 min at 37°C. Phosphate release from GMP is plotted as a function of magnesium concentration. Each datum is the average of three independent experiments ± SEM. (**D**) Enzyme titration. Reaction mixtures (50 µL) containing 50 mM Tris-HCl, pH 7.0, 1 mM GMP, 5 mM MgCl_2_, 0.1% (wt/vol) BSA, and Ifn1 as specified (in pmol) were incubated for 30 min at 37°C. Phosphate release from GMP is plotted as a function of input Ifn1. Each datum is the average of three independent experiments ± SEM. (**E**) Temperature dependence. Reaction mixtures (50 µL) containing 50 mM Tris-HCl, pH 7.0, 1 mM GMP, 5 mM MgCl_2_, 0.1% (wt/vol) BSA, and 15 ng Ifn1 were incubated for 30 min at 24°C, 30°C, 37°C, 42°C, or 55°C. Phosphate release is plotted in bar graph format for each condition tested. Bar height is the average of three independent experiments ± SEM, with individual replicates indicated by filled circles. (**F**) Reaction rate. A reaction mixture (350 µL) containing 50 mM Tris-HCl, pH 7.0, 1 mM GMP, 5 mM MgCl_2_, 0.1% (wt/vol) BSA, and 105 ng Ifn1 was incubated at 37°C. Aliquots (50 µL) were withdrawn at the times specified and quenched. The extent of phosphate release is plotted as a function of time. Each datum is the average of three independent experiments ± SEM. (**G and H**) Nucleotide substrate specificity. Reaction mixtures (50 µL) containing 50 mM Tris-HCl, pH 7.0, 5 mM MgCl_2_, 1 mM of the indicated nucleotide substrate, 0.1% (wt/vol) BSA, and 15 ng Ifn1 were incubated for 30 min at 37°C. The extents of phosphate release from each substrate are shown. Bar height is the average of three independent experiments ± SEM, with individual replicates indicated by filled circles.

Activity at 37°C was optimal at pH 6.5–7.0 and declined progressively as pH was lowered to 6.0 (to 43% of activity at pH 7.0) and 5.5 (11% activity) or increased to 8.0 (34%) and 9.0 (9%). Ifn1 was inactive at pH 5.0 and 9.5 (Fig. 2A). The bell-shaped pH profile suggests that activity requires at least one moiety to be deprotonated and at least one moiety to be protonated on the enzyme (or the GMP substrate).

Ifn1 activity was strictly dependent on an added divalent cation (Fig. 2B). A comparison of various metal ions at 2 mM concentration (each as the chloride salt, except CdSO_4_) showed that Mg^2+^ was the preferred cofactor, followed by Co^2+^, Mn^2+^, and Ni^2+^. By contrast, Zn^2+^, Cu^2+^, Cd^2+^, and Ca^2+^ were ineffective (Fig. 2B). We proceeded to conduct a metal mixing experiment in which Ifn1 reactions containing 2 mM Mg^2+^ were supplemented with 2 mM of another divalent cation. We found that Zn^2+^, Cu^2+^, Cd^2+^, and Ca^2+^ inhibited Ifn1 activity in the presence of Mg^2+^ (Fig. 2B). We speculate that the inhibitory metal ions outcompete magnesium for occupancy of a divalent cation site on the Ifn1-GMP complex. When engaged, they are unable to support phosphohydrolase reaction chemistry. A Mg^2+^ titration experiment showed that GMP hydrolysis varied linearly with Mg^2+^ concentration up to 2 mM and increased further at 5 and 10 mM (Fig. 2C). Ensuing assays were routinely performed at 5 mM Mg^2+^.

The yield of free phosphate during a 30-min reaction increased with reaction temperature in the range of 24°C–42°C before declining acutely at 55°C (Fig. 2E). Product formation at 37°C increased with input Ifn1, reaching saturation at a point where 88% of the input GMP was hydrolyzed in 30 min (Fig. 2D). From the slope of the titration curve in the linear range, we calculated that 28.8 ± 0.8 nmol of product was formed per picomole of Ifn1, which corresponds to an estimated turnover number of 16 s^-1^. Phosphate release from 1 mM GMP increased linearly with time over 60 min (Fig. 2F). From a linear regression fit of the data, we derived a turnover number of 16.7 s^-1^.

### Nucleotide substrate specificity

To test NMP preference, Ifn1 was reacted for 30 min at 37°C with either 1 mM GMP, AMP, CMP, or UMP. GMP was the best substrate, followed by CMP, UMP, and AMP, which were hydrolyzed to the extents of 58%, 31%, and 9%, respectively, of that seen with GMP (Fig. 2G). We detected no phosphate release from 1 mM NMN or NAD^+^ (Fig. 2G). These results show that Ifn1 differs in its substrate specificity from the budding yeast homolog Sdt1. Ifn1 apparently requires a ribose sugar, insofar as hydrolysis of dGMP was 3% that of GMP, and hydrolysis of dCMP was 10% that of CMP (Fig. 2G). Ifn1 was inactive with 1 mM 3′-GMP or 3′-CMP (Fig. 2G). Ifn1 was unable to release phosphate from 1 mM GTP; activity with 1 mM GDP was 11% that of GMP (Fig. 2H).

### Structural modeling of Ifn1

**Fig 3:**
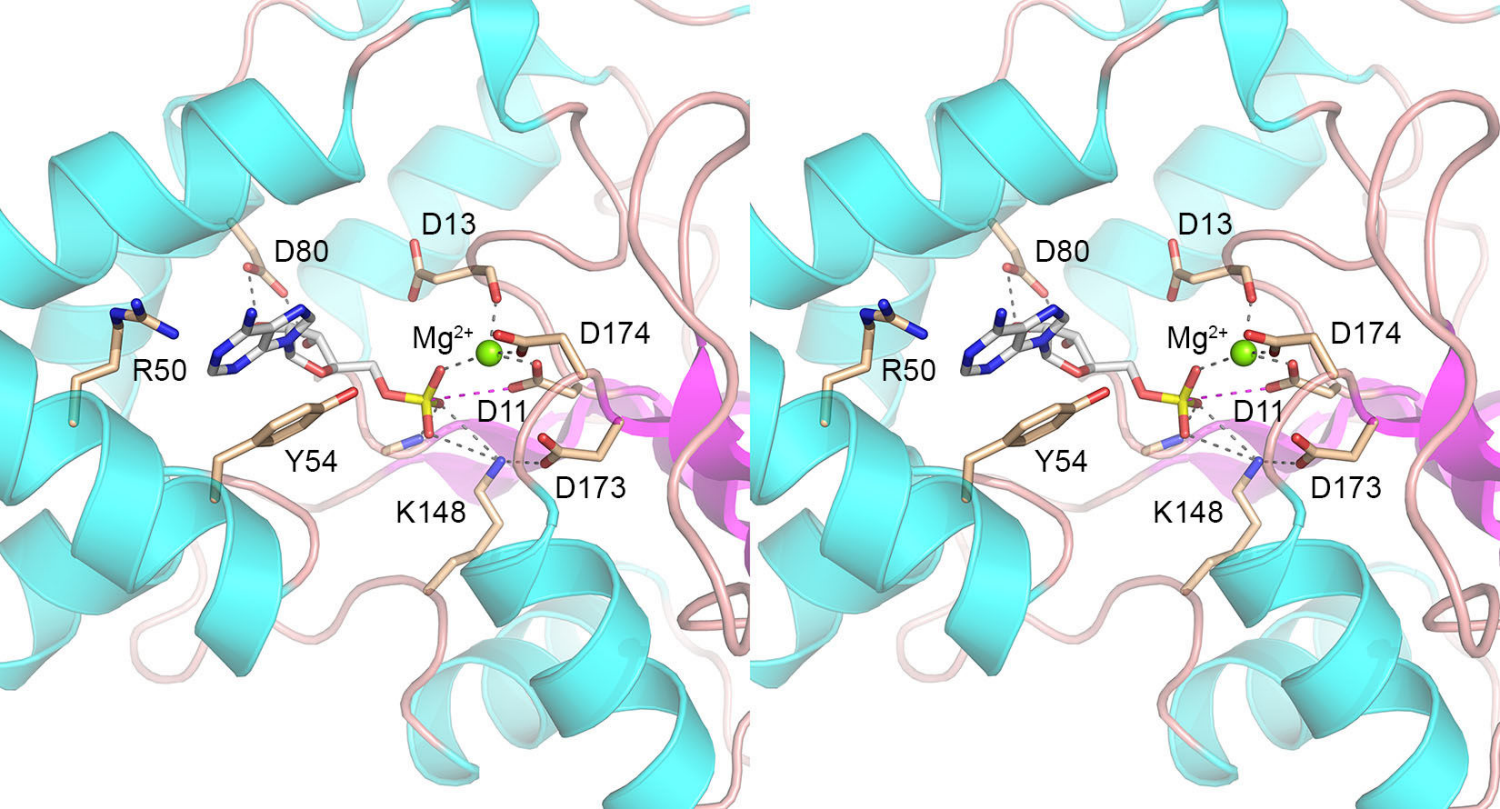
AlphaFold 3 model of Ifn1 active site in complex with Mg^2+^ and AMP. Stereo view of the Ifn1 active site containing a magnesium ion (green sphere) and AMP (stick model with gray carbons). The polypeptide is depicted as a cartoon trace with cyan α-helices and magenta β-strands, with selected amino acids rendered as stick models with beige carbons. Atomic contacts are denoted by dashed lines.

We exploited the AlphaFold 3 server (16) to generate a model of Ifn1 in complex with Mg^2+^ and AMP. (GMP, CMP, and UMP are not actionable ligands on the AlphaFold 3 server.) A stereo view of the predicted Ifn1 active site is shown in Fig. 3. The magnesium ion (green sphere) is coordinated by Asp11-Oδ2, Asp174-Oδ1, the Asp13 mainchain carbonyl, and an NMP phosphate oxygen. The remaining positions in the expected octahedral Mg^2+^ complex are presumably occupied by water molecules. Asp11-Oδ1 is situated 3.6 Å from the NMP phosphorus in a favorable orientation for nucleophilic attack of the phosphate and expulsion of the guanosine (Oδ1–P–O5′ angle of 156˚). The NMP phosphate oxygens are coordinated by Lys148-Nζ, which is held in position by a salt bridge to Asp173. The phosphate also receives a hydrogen bond from the Asn113 mainchain amide. The phosphate oxygen contacts the enzyme and Mg^2+^, presumably stabilizing the transition state of the phosphoryl transfer reaction. The model provides several insights into substrate binding and specificity. To wit: (i) Tyr54 makes a π-stack on the nucleobase of the 5′-NMP; (ii) bidentate hydrogen bonds from the Asp80 carboxylate to the ribose 2′-OH and 3′-OH may account for the ribonucleotide specificity of Ifn1, as well as the failure to hydrolyze nucleoside-3′-monophosphates. In addition, Arg50, which is adjacent to the modeled adenine, is a potential determinant of the preference of Ifn1 for GMP over AMP, insofar as this arginine is poised to make a hydrogen bond to guanine-O6 that would not be compatible with adenine-N6.

### Asp11, Asp80, and Asp174 are essential for 5′-nucleotidase activity

**Fig 4:**
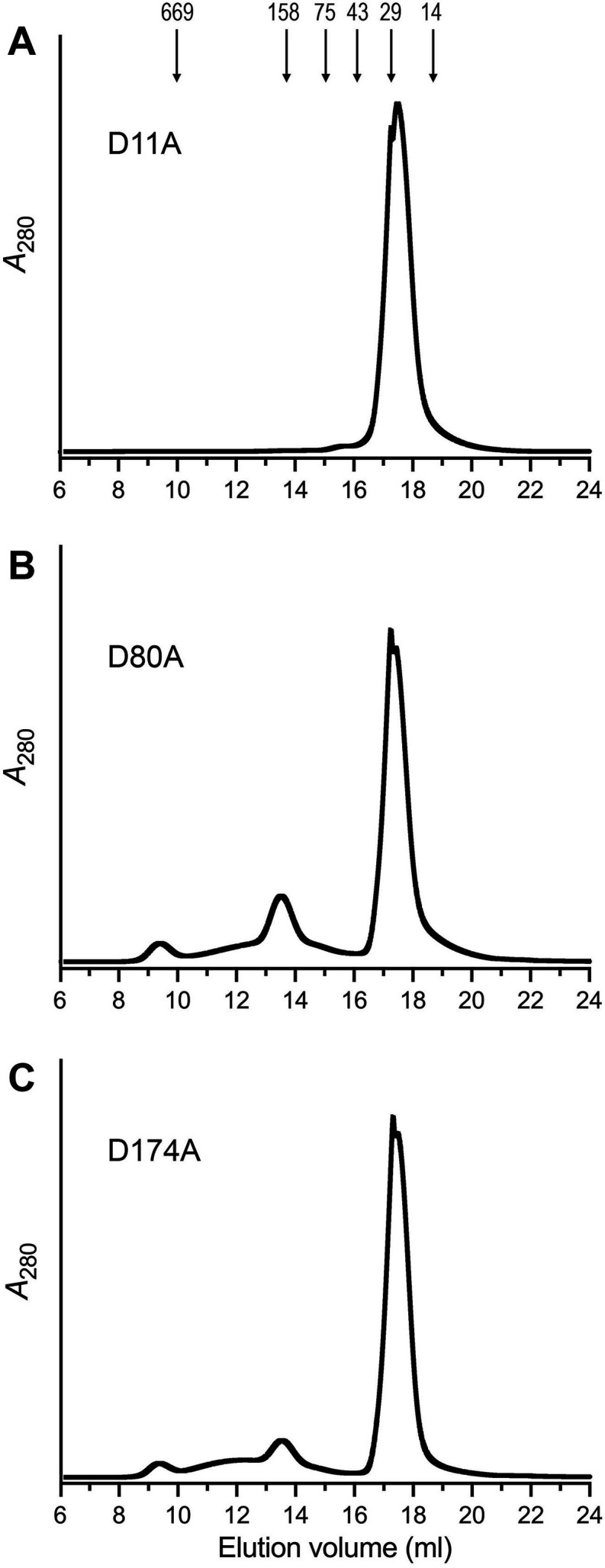
Gel filtration of Ifn1 mutants D11A (**A**), D80A, (**B**), and D174A (**C**). Tag-free Ifn1 mutants D11A, D80A, and D174A were gel filtered through a 24 mL Superdex-200 column. The elution profiles were monitored continuously by *A*_280_ as a function of elution volume.

**Fig 5:**
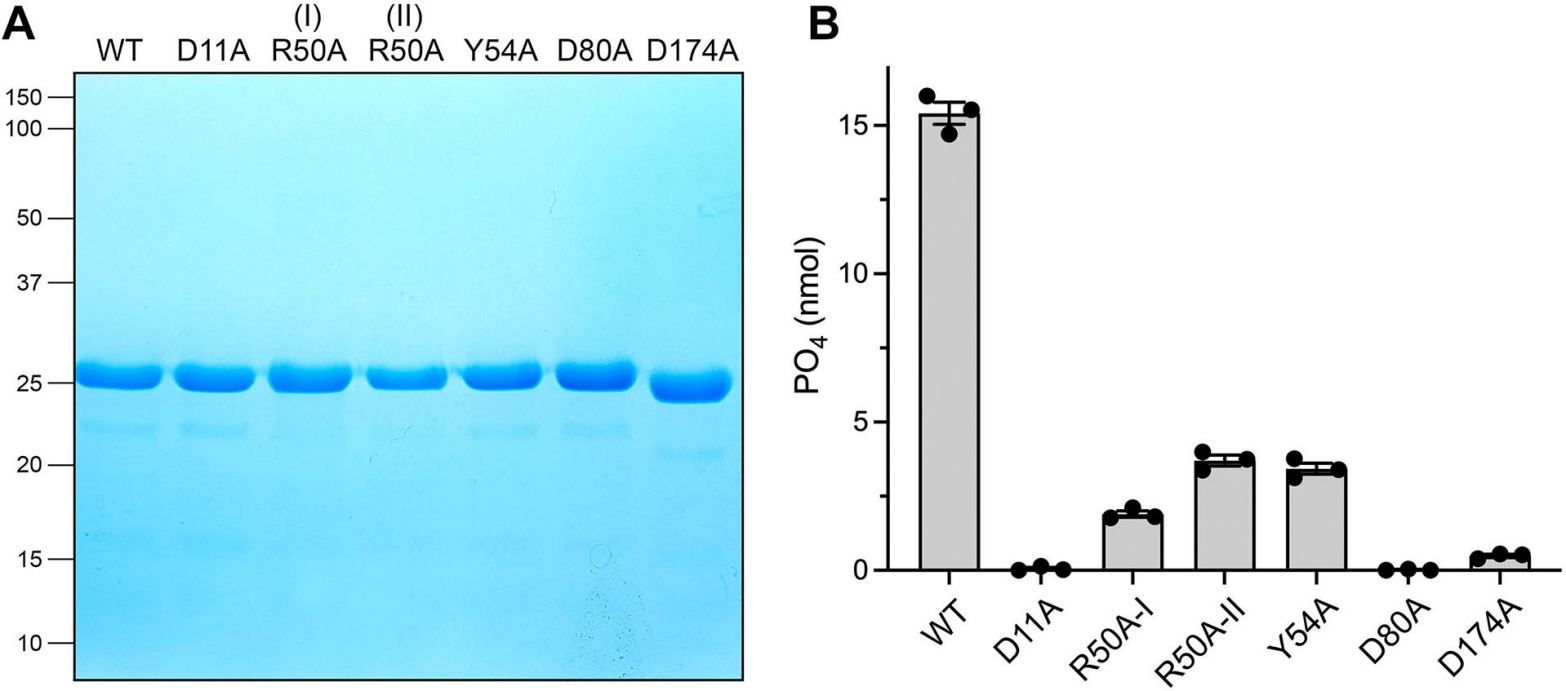
Structure-guided mutagenesis. (**A**) Aliquots (10 µg) of the peak Superdex-200 fractions of the indicated recombinant Ifn1 preparations were analyzed by SDS-PAGE. The Coomassie blue-stained gel is shown, with the positions and sizes (kDa) of marker polypeptides indicated on the left. (**B**) Reaction mixtures (50 µL) containing 50 mM Tris-HCl, pH 7.0, 5 mM MgCl_2_, 1 mM GMP, 0.1% (wt/vol) BSA, and 15 ng wild-type or mutant Ifn1 as specified were incubated for 30 min at 37°C. The extents of phosphate release are shown. Bar height is the average of three independent experiments ± SEM, with individual replicates indicated by filled circles.

Single alanine substitutions were introduced in lieu of Asp11, Asp80, and Asp174, and the recombinant Ifn1-Ala proteins were produced in *E. coli* and purified as described for wild-type Ifn1. The D11A protein gel-filtered as a single monomeric peak (Fig. 4A), whereas the majority of the D80A and D174A preparations also eluted as monomeric proteins during gel filtration. A minor fraction of the D80A and D174A Ifn1 polypeptides comprised a second heavier peak (at elution volumes of 13.5 mL), suggestive of an oligohexameric Ifn1 complex (Fig. 4B and C). (SDS-PAGE of the minor peak fractions revealed only the Ifn1 polypeptide [not shown].) The monomeric D11A, D80A, and D174A preparations, which were of equivalent purity (Fig. 5A), were assayed for GMP hydrolysis in parallel with wild-type Ifn1. The D11A and D80A mutants displayed no detectable activity at a level of enzyme that sufficed for hydrolysis of 30% of the input GMP by wild-type Ifn1 (Fig. 5B). Hydrolysis of GMP by the D174A mutant was 3% that seen with wild-type Ifn1 (Fig. 5B). We conclude that these three aspartates are essential for 5′-nucleotidase activity by virtue of their imputed roles in covalent phosphoryl transfer (Asp11), engagement of the ribose hydroxyls (Asp80), and coordination of the magnesium cofactor (Asp174).

### Alanine mutations at Arg50 and Tyr54

**Fig 6:**
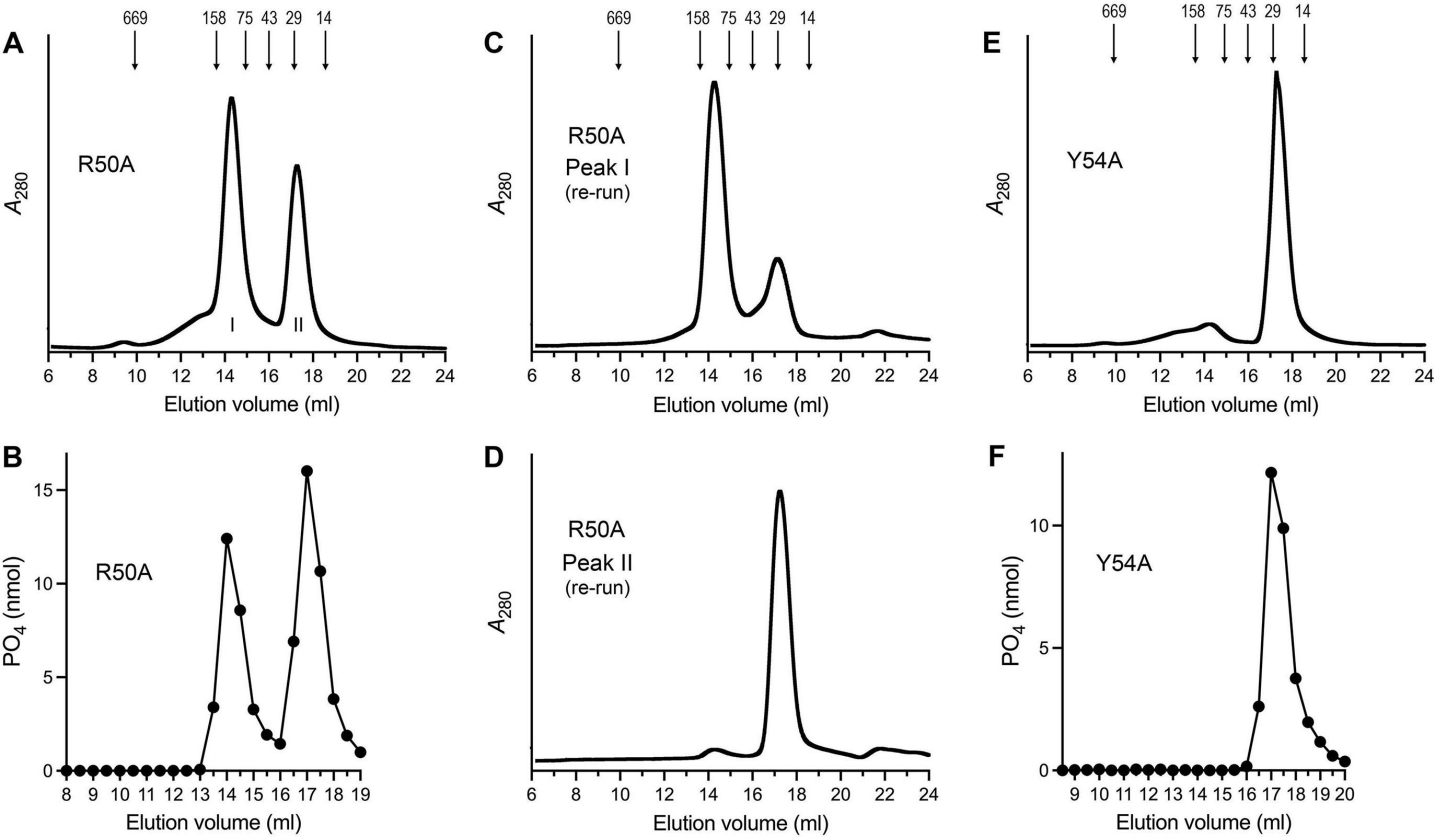
Gel filtration of Ifn1 mutants R50A and Y54A. (**A**) Tag-free Ifn1-R50A was gel filtered through a 24 mL Superdex-200 column. The elution profile was monitored continuously by *A*_280_ as a function of elution volume. (**B**) Reaction mixtures (50 µL) containing 50 mM Tris-HCl, pH 7.0, 5 mM MgCl_2_, 1 mM GMP, 0.1% (wt/vol) BSA, and equal volume aliquots of serial R50A Superdex-200 fractions were incubated for 30 min at 37°C. Phosphate release from GMP is plotted as a function of elution volume. (**C and D**) The R50A Superdex-200 fraction corresponding to peak I and peak II in panel A was individually subjected to a second round of gel filtration. The elution profiles are shown. (**E**) Tag-free Ifn1-Y54A was gel filtered through a 24 mL Superdex-200 column. The elution profile is shown. (**F**) Reaction mixtures (50 µL) containing 50 mM Tris-HCl, pH 7.0, 5 mM MgCl_2_, 1 mM GMP, 0.1% (wt/vol) BSA, and equal volume aliquots of serial Y54A Superdex-200 fractions were incubated for 30 min at 37°C. Phosphate release is plotted as a function of elution volume.

We also introduced alanine in lieu of Arg50 and Tyr54, which are implicated by AlphaFold in contact with the nucleobase of the 5′-NMP substrate. The R50A mutant eluted as two discrete peaks during gel filtration: (i) a heavier component (peak I in Fig. 6A; elution volume 14.3 mL, intermediate between the 158 and 75 kDa markers) consisting of the Ifn1 polypeptide (Fig. 5A) that we surmise is an Ifn1-R50A homotetramer; and (ii) a lighter component (peak II in Fig. 6A) corresponding to an Ifn1-R50A monomer (Fig. 5A). Assay of equal volume aliquots of serial column fractions for GMP hydrolysis showed that both peaks were catalytically active (Fig. 6B), with the monomer peak displaying higher activity when normalized for *A*_280_. When assayed in parallel with wild-type Ifn1, the extents of GMP hydrolysis by the R50A peak I and II proteins were 12% and 24%, respectively, of the wild-type activity (Fig. 5B). Subjecting R50A peak I to a second round of gel filtration revealed that while the majority of the protein continued to elute as a putative tetramer, a minority dissociated and eluted as a monomer (Fig. 6C). By contrast, virtually all of the R50A peak I protein continued to elute as a monomer during a second round of gel filtration (Fig. 6D).

The majority of the Ifn1-Y54A mutant protein, and all of the 5′-nucleotidase activity, eluted as a monomer during gel filtration (Fig. 6E and F). The Y54A mutant was 22% as active as wild-type Ifn1 in hydrolyzing GMP (Fig. 5B).

### Arg50 is a determinant of nucleotide substrate preference

**Fig 7:**
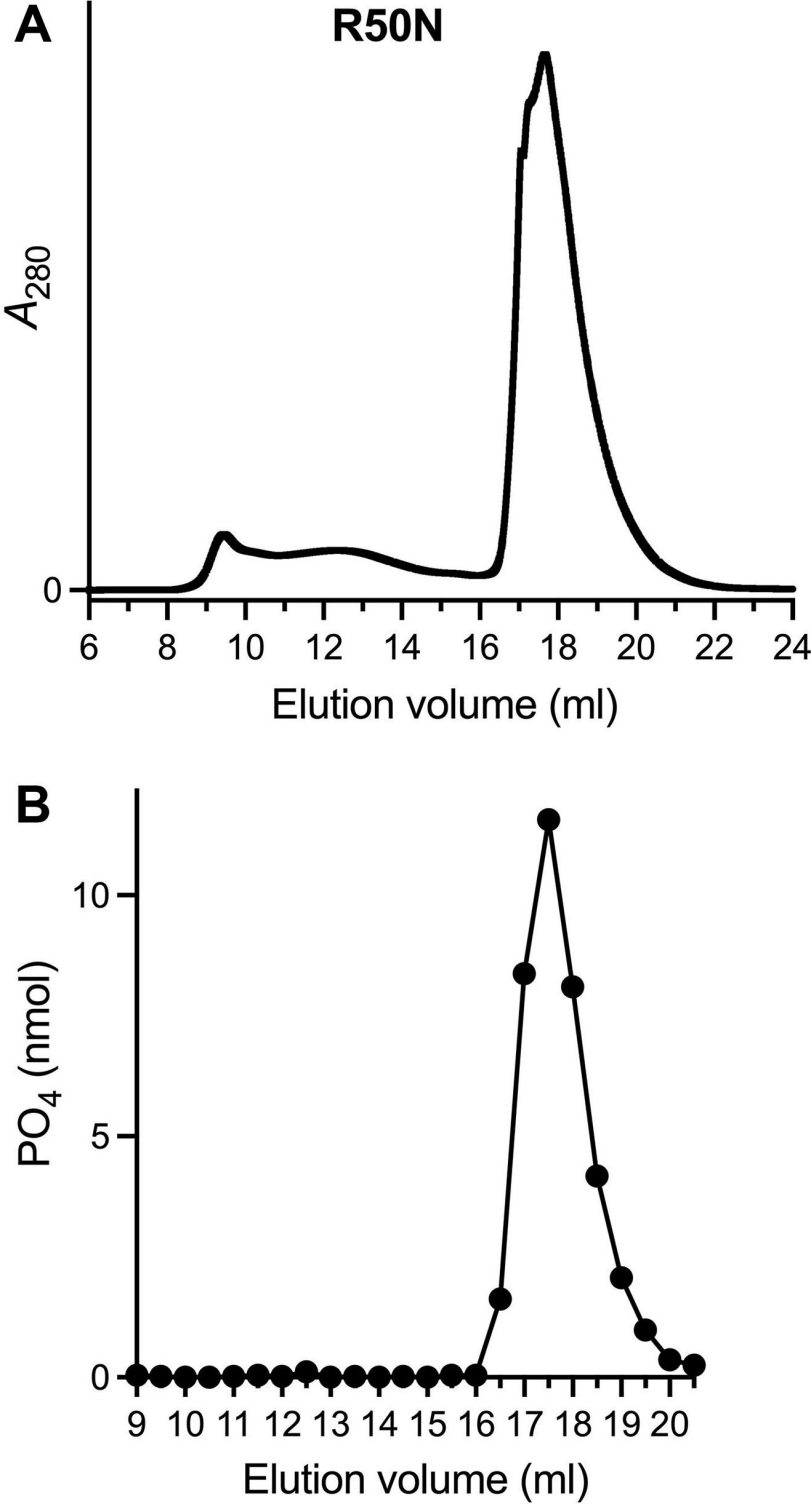
Gel filtration of Ifn1 mutant R50N. (**A**) Tag-free Ifn1-R50N was gel filtered through a 24 mL Superdex-200 column. The elution profile was monitored continuously by *A*_280_ as a function of elution volume. (**B**) Reaction mixtures (50 µL) containing 50 mM Tris-HCl, pH 7.0, 5 mM MgCl_2_, 1 mM CMP, 0.1% (wt/vol) BSA, and equal volume aliquots of serial R50N Superdex-200 fractions were incubated for 30 min at 37°C. Phosphate release from CMP is plotted as a function of elution volume.

**Fig 8:**
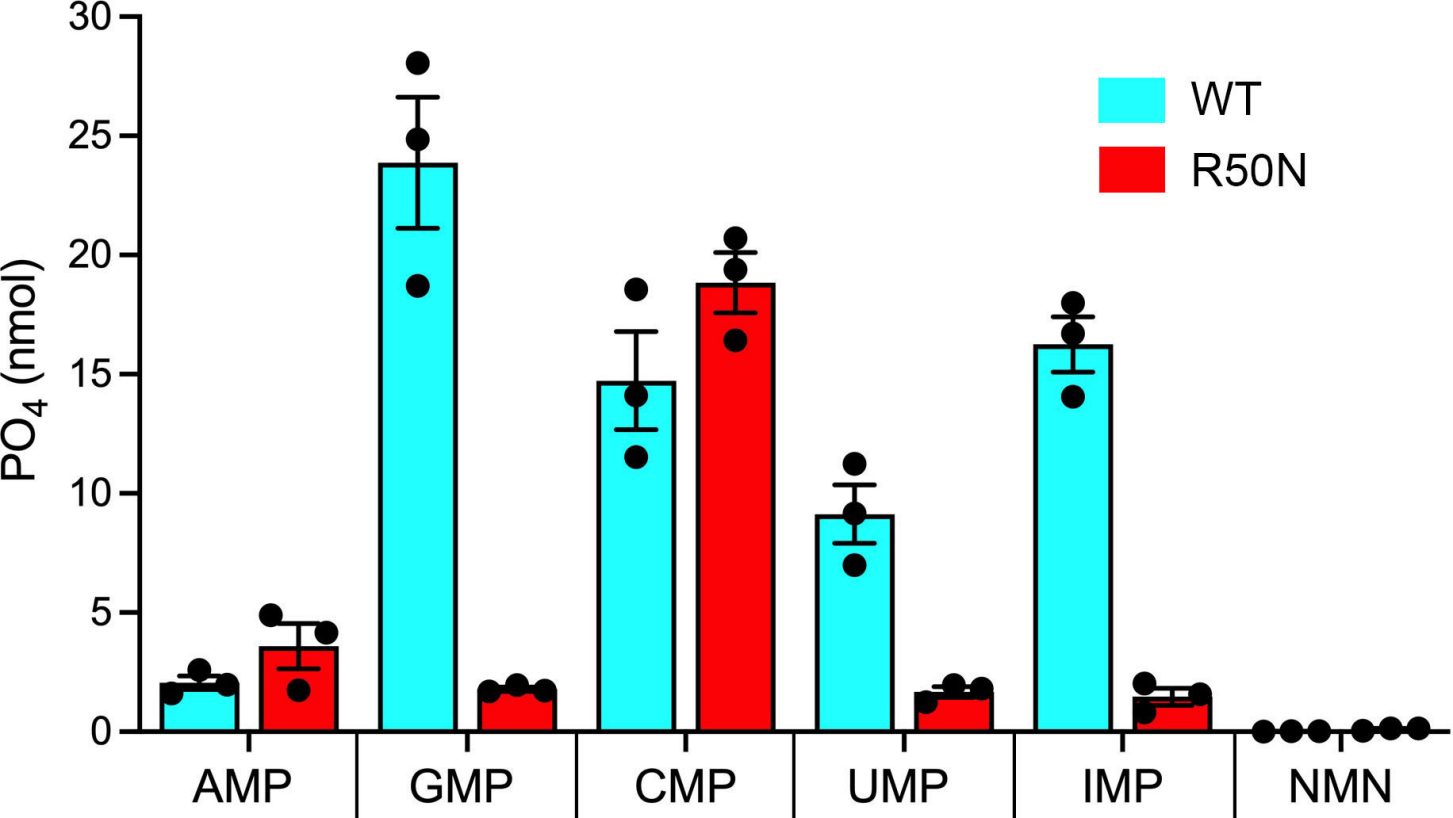
R50N elicits a change in Ifn1 substrate specificity in favor of CMP. Reaction mixtures (50 µL) containing 50 mM Tris-HCl, pH 7.0, 5 mM MgCl_2_, 1 mM of the indicated nucleotide substrate, 0.1% (wt/vol) BSA, and 15 ng wild-type Ifn1 or mutant R50N were incubated for 30 min at 37°C. The extents of phosphate release from each substrate are shown. Bar height is the average of three independent experiments ± SEM, with individual replicates indicated by filled circles.

As noted above, the proximity of Arg50 to the nucleobase in the AlphaFold model suggests a role in substrate selectivity. The equivalent amino acid in Sdt1 is asparagine. To query whether a single amino acid difference might explain the distinctive substrate specificities of Ifn1 and Sdt1, we changed Ifn1 Arg50 to Asn and purified the recombinant R50N protein, which eluted as a monomer during gel filtration (Fig. 7A). Because pilot experiments indicated that CMP was the preferred NMP substrate for the R50N enzyme, we assayed CMP hydrolysis by equal volume aliquots of serial column fractions and found that nucleotidase activity tracked with the monomeric R50N protein (Fig. 7B). To evaluate the impact of the R50N change on substrate specificity, the R50N mutant and an equivalent amount of wild-type Ifn1 were reacted in parallel for 30 min at 37°C with either 1 mM GMP, IMP, AMP, CMP, UMP, or NMN (Fig. 8). The wild-type substrate preference echoed that shown in Fig. 2G, with the added insight that IMP was hydrolyzed 68% as well as GMP, making it the second-best substrate of those tested. The salient findings were that the R50N mutation elicited a gain of activity with CMP (to 128% of wild type) and AMP (to 128% of wild type) and a suppression of activity with GMP (13% of wild type), IMP (9% of wild type), and UMP (13% of wild type) (Fig. 8). The hierarchy of substrate usage by R50N was CMP ≫ AMP > GMP/UMP/IMP. Note that the R50N mutation did not confer any activity with NMN (Fig. 8).

### Ifn1 overexpression is toxic in fission yeast

**Fig 9:**
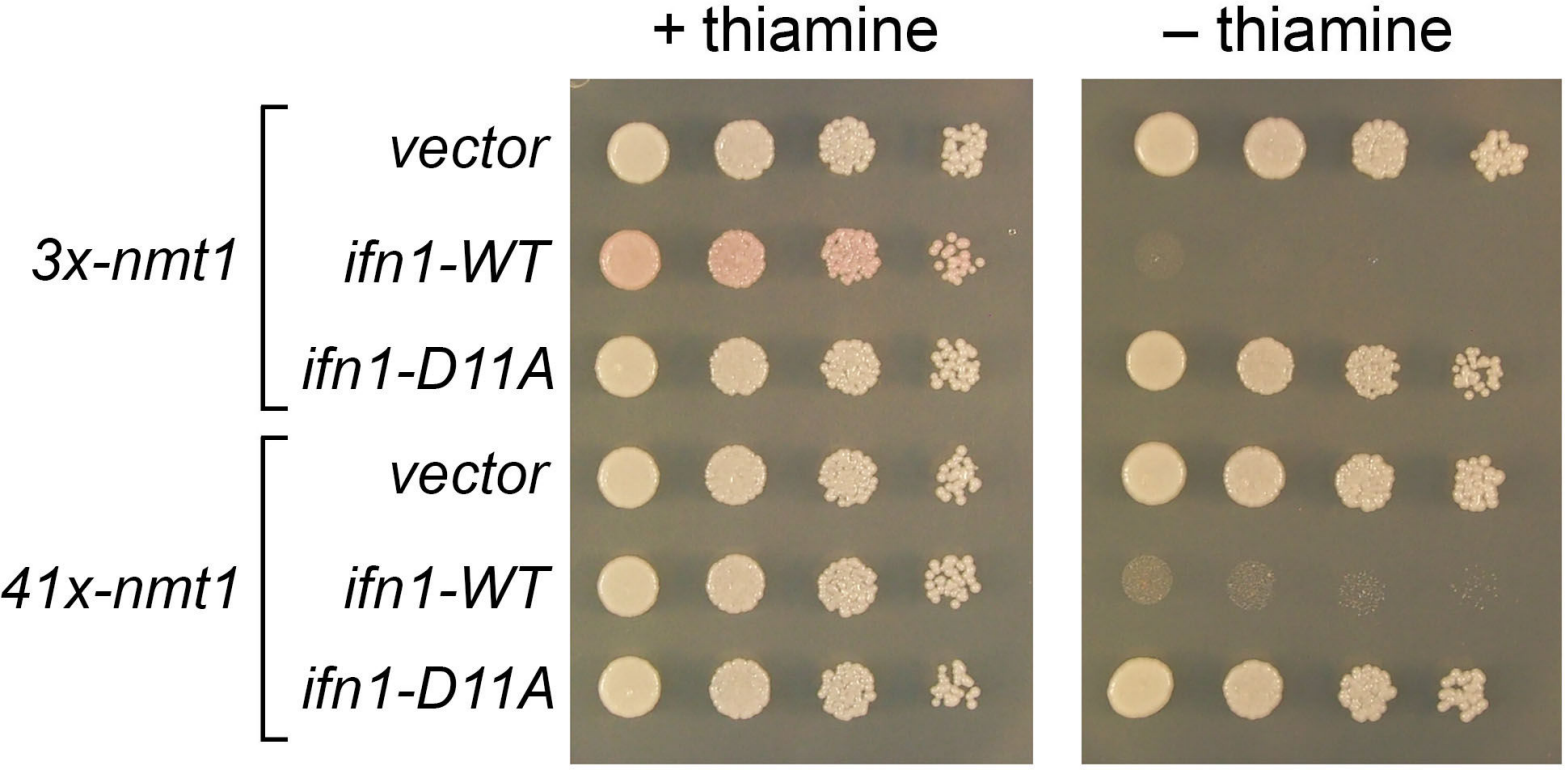
Effect of Ifn1 overexpression. Serial dilutions of fission yeast cells bearing the indicated plasmids expressing wild-type Ifn1 or Ifn1-D11A (as specified on the left) were spot tested for growth on ePMGT(−leu) agar (+thiamine) or ePMG(−leu) agar (–thiamine) in parallel with empty vector controls. The plates were photographed after 4 days of incubation at 30°C.

The *ifn1*^+^ gene is inessential for vegetative growth on YES medium, and *ifn1*∆ cells display normal morphology (17). To gauge the impact of overexpressing Ifn1, we placed the *ifn1* ORF, either *ifn1-WT* or *ifn1-D11A*, on a multicopy plasmid under the control of the thiamine-repressible *3x-nmt1* high-strength promoter or the *41x-nmt1* medium-strength promoter. Cells bearing the *ifn1* plasmids, or the corresponding empty vectors, were grown in liquid medium containing thiamine, and serial dilutions were spotted on agar medium containing thiamine (expression OFF) or agar medium lacking thiamine (expression ON). The *ifn1-WT* plasmids elicited no effect on growth in the presence of thiamine but were toxic in the absence of thiamine (Fig. 9). By contrast, *3x-nmt1* and *41x-nmt1* plasmids expressing *ifn1-D11A* were non-toxic on medium lacking thiamine (Fig. 9), signifying that the inhibition of fission yeast growth upon Ifn1 overexpression is caused by excess 5′-nucleotidase activity. It was noteworthy that cells bearing the *3x-nmt1 ifn1* plasmid displayed a pink color under non-inducing conditions (+thiamine) that was not evident in cells bearing the empty *3x-nmt1* vector or the *3x-nmt1* plasmid expressing *ifn1-D11A* (Fig. 9). As discussed below, accumulation of pink pigment in yeast cells is a feature of aberrant *de novo* purine biosynthesis.

## DISCUSSION

Here, we characterized the phosphate starvation-induced fission yeast Ifn1 protein as a Mg^2+^-dependent 5′-nucleotidase of the HAD aspartyl-phosphatase superfamily. Unlike its budding yeast homolog Sdt1, Ifn1 displays a distinctive preference for hydrolysis of GMP and IMP and is unable to hydrolyze NMN (a signature property of Sdt1). Although not annotated as such in Pombase, Ifn1 is also homologous to a second budding yeast aspartyl-phosphatase 5′-nucleotidase enzyme, Phm8, a 321-aa polypeptide with 127 positions of identity/similarity to Ifn1. The N-terminal 47-aa segment of Phm8 has no counterpart in Ifn1. The *PHM8* gene is transcriptionally upregulated in response to phosphate starvation, and screening of the recombinant Phm8 protein for hydrolysis of 90 phosphorylated compounds pinpointed nucleoside-5′-monophosphates as the best substrates, with a strong preference for CMP and XMP (xanthosine monophosphate) followed by (in decreasing order of specific activity) GMP, UMP, AMP, and IMP (18). The specific activity of Phm8 with IMP was at least an order of magnitude lower than with CMP (18). Thus, the substrate preferences of fission yeast Ifn1 are distinct from either of the budding yeast homologs.

A targeted alanine scan of the Ifn1 active site, guided by an AlphaFold model of a Mg^2+^•NMP Michaelis complex, identified essential catalytic residues and revealed determinants of NMP specificity. The results affirmed Asp11 as the nucleophile, highlighted Asp174 as a key metal-ligand, and implicated Asp80 in specificity for ribonucleotides via its engagement of the ribose hydroxyls. Whereas the D11A, D174A, and D80A mutations effectively abolished GMP hydrolysis, activity was merely reduced several-fold when Tyr54 and Arg50 were replaced by alanine. This is consistent with the model predictions that Tyr54 and Arg50 are poised to interact with the NMP nucleobase via a π-stack (Tyr54) and (potentially) hydrogen bonding (Arg50), respectively, and are at a distance from the site at which chemistry occurs. We envision that the Y54A and R50A mutations reduce affinity for the GMP substrate.

The most compelling finding was that a single swap of Ifn1 Arg50 to Asn (the equivalent position in Sdt1) elicited a substrate specificity switch evinced by a gain of activity with CMP and AMP and suppression of activity with GMP, IMP, and UMP. We hypothesize that Arg50 confers a preference for GMP and IMP over AMP by virtue of hydrogen bond donation to the nearby O6 atom of guanine/hypoxanthine, which would not be allowed to adenine-N6. That said, the low activity observed for wild-type Ifn1 with AMP might be aided by hydrogen bond donation from Arg50 to adenine-N1, which would not apply to the protonated guanine/hypoxanthine-N1 atom. Asparagine in lieu of arginine could facilitate a new bidentate hydrogen bonding arrangement whereby Asn-Oδ and Asn-Nδ engage adenine-N6 and adenine-N1, respectively, which would account for the observation that AMP hydrolysis by R50N is maintained and is even slightly higher than that of wild-type Ifn1. In principle, simply flipping the Asn50 amide rotamer could allow Asn-Oδ and Asn-Nδ to hydrogen bond with guanine-N1 and -O6, respectively. However, there may be constraints on this arrangement, insofar as R50N activity with GMP/IMP is much lower than wild-type Ifn1, and R50N is more active with AMP than GMP/IMP. We suggest that the strong preference of R50N for CMP hydrolysis may reflect a new bidentate hydrogen bonding pattern in which Asn-Nδ and Asn-Oδ engage cytosine-N3 and -N4, respectively. Although a definitive interpretation of the results will hinge on determining the structures of wild-type Ifn1 and the R50N mutant in complexes with the various NMP nucleotides, the emergent theme is that 5′-nucleotidase substrate specificity is a tunable property.

Inducible high-level expression of the *ifn1*^+^ gene is highly toxic to fission yeast, and the toxicity is contingent on Ifn1 catalytic activity. These findings echo the report that galactose-induced overexpression of catalytically active Sdt1 inhibited the growth of *S. cerevisiae* (15). A strong clue that Ifn1 affects purine nucleotide metabolism *in vivo* stems from the finding that basal expression of active Ifn1 from the multicopy *3x nmt1* plasmid elicits the accumulation of pink pigment in the absence of a growth defect. *De novo* purine biosynthesis is regulated by feedback inhibition, whereby the end products IMP, GMP, and AMP inhibit the rate-limiting enzyme glutamine:PRPP aminotransferase (Ade4 in fission yeast) at the beginning of the pathway. Mutations in the downstream enzyme phosphoribosylaminoimidazole carboxylase (Ade6) cause accumulation of the intermediate phosphoribosylaminoimidazole, which is converted to form a red pigment. Our “wild-type” *S. pombe* strain bears a hypomorphic *ade6-M216* (Gly16Asp) missense mutation that confers a pink colony color on adenine-limited medium (19). However, the *ade6-M216* strain forms white colonies on ePMGT medium that contains 0.25 g/L of adenine. A gain of pink colony pigment was seen when basal Ifn1 expression stemmed from the highest strength *3x-nmt1* promoter but not from the intermediate strength *41x-nmt1* promoter (Fig. 9). This suggests that elevated basal Ifn1 reduces the cellular pool of IMP/GMP (its preferred substrates) and thereby enhances flux through the *de novo* purine synthesis pathway by relieving IMP/GMP-mediated feedback inhibition.

**Fig 10:**
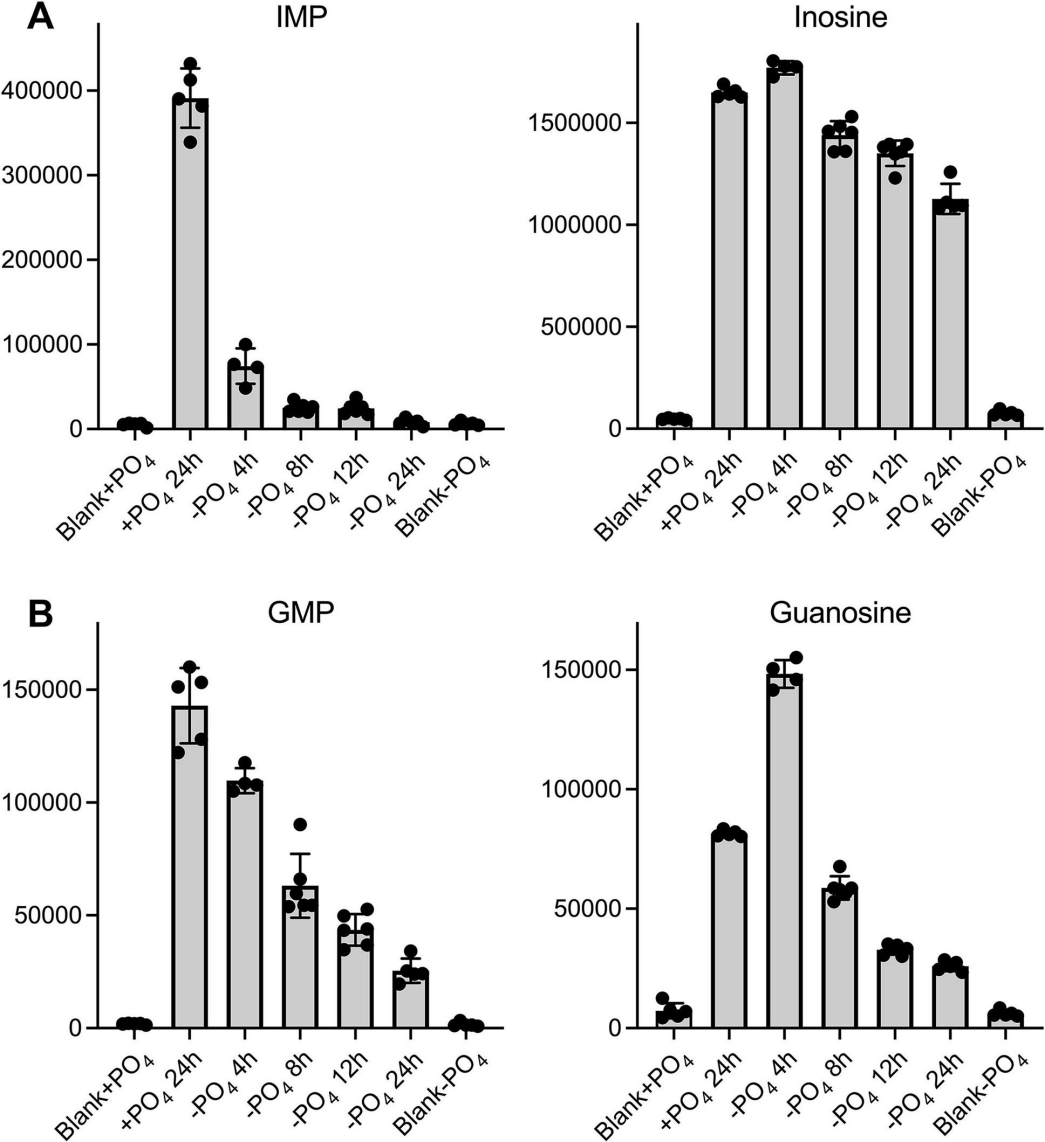
Depletion of inosine/guanine NMP pools during phosphate starvation. The bar graphs show the levels of the IMP and inosine (**A**), and GMP and guanosine (**B**) in fission yeast cells grown in phosphate-replete medium (+PO4 24 h) and cells maintained in phosphate-free medium (–PO4) for 4, 8, 12, and 24 h, as indicated on the *x*-axis (see reference 7 for full details). The LC/MS peak areas for the individual biological replicates are denoted by filled circles. Each bar height is the mean of the individual peak areas ± SD. The +PO4 and –PO4 medium-only blank values are indicated in the leftmost and rightmost bars.

Finally, it is worth pointing out that our previous metabolomics survey of phosphate-starved fission yeast (7) revealed that the intracellular pool of IMP was rapidly depleted as a function of starvation time, while the GMP pool also declined, albeit more gradually (Fig. 10). The pool of inosine was maintained at pre-starvation levels, while the pool of guanosine increased during acute phosphate starvation before declining during the chronic phase (Fig. 10). It is likely that 5′-nucleotidases are contributory to the increased NMP catabolism that accompanies phosphate starvation of fission yeast. Besides Ifn1, four other predicted fission yeast intracellular NMP 5′-phosphatase enzymes are transcriptionally upregulated during phosphate starvation (2), including Isn1, which is annotated in Pombase as an IMP-specific 5′-nucleotidase. In light of the present study, it is evident that such functional predictions, especially regarding substrate specificity, need to be interrogated directly for each enzyme, as a prelude to better understanding NMP dynamics during phosphate starvation.

## MATERIALS AND METHODS

### Recombinant Ifn1

The ORF encoding Ifn1 was PCR-amplified from *S. pombe* genomic DNA with primers that introduced a BamHI site immediately flanking the Val3 codon and an XhoI site downstream of the stop codon. The PCR product was digested with BamHI and XhoI and inserted between the BamHI and XhoI sites of pET28b-His_10_Smt3 to generate a T7 RNA polymerase-based expression plasmid encoding Ifn1-(3-226) fused to an N-terminal His_10_Smt3 tag. Plasmids expressing Ifn1 missense mutants D11A, D80A, D174A, Y54A, R50A, and R50N were constructed by inverse PCR and In-Fusion cloning (https://www.takarabio.com/learning-centers/cloning/applications-and-technical-notes/mutagenesis-with-in-fusion-cloning). All plasmid inserts were sequenced to exclude the presence of unwanted mutations. The pET28b-His_10_Smt3-Ifn1 plasmids were transformed into *E. coli* BL21(DE3) cells. Cultures (1,000 mL) amplified from single kanamycin-resistant transformants were grown at 37°C in Luria-Bertani medium containing 50 μg/mL kanamycin until the *A*_600_ reached ~0.6. The cultures were chilled on ice for 1 h, adjusted to 2% (vol/vol) ethanol and 0.5 mM IPTG, and then incubated for 16–18 h at 17°C with constant shaking. Cells were harvested by centrifugation and stored at −80°C. All subsequent steps were performed at 4°C. Cells were thawed and resuspended in 25 mL of buffer A (50 mM Tris-HCl, pH 8.0, 500 mM NaCl, 20 mM imidazole, 1 mM DTT, and 20% glycerol) containing 1 mg/mL lysozyme and 1/2 tablet of EDTA-free protease inhibitor cocktail (ThermoFisher). After incubation for 45–60 min, the lysates were sonicated to reduce viscosity, and the insoluble material was removed by centrifugation at 18,000 rpm for 35 min. The supernatants were mixed overnight with 2 mL of Ni-nitrilotriacetic acid agarose resin (Qiagen) that had been equilibrated with buffer A. The resin was recovered by centrifugation and washed twice with 40 mL of buffer A. The resin was centrifuged again, resuspended in 20 mL of buffer B (50 mM Tris-HCl, pH 8.0, 500 mM NaCl, 1 mM DTT, and 10% glycerol) with 20 mM imidazole, and poured into a column. After washing the column sequentially with 8 mL of 3 M KCl, 20 mL of buffer B with 20 mM imidazole, and 10 mL of buffer B with 50 mM imidazole, the bound material was eluted with 10 mL of buffer B containing 500 mM imidazole while collecting 1 mL fractions. The polypeptide compositions of the flow-through and eluate fractions were monitored by SDS-PAGE. The 500 mM imidazole eluate fractions containing His_10_Smt3-Ifn1 were supplemented with 10 µg of Smt3-specific protease Ulp1 and then dialyzed overnight against 1,000 mL of buffer C (20 mM Tris-HCl, pH 8.0, 250 mM NaCl, 20 mM imidazole, 1 mM DTT, and 10% glycerol), during which time the His_10_Smt3 tag was cleaved. The dialysates were mixed for 30 min with 2 mL of Ni-nitrilotriacetic acid agarose resin that had been equilibrated with buffer C. Tag-free Ifn1 proteins were recovered in the flow-through fractions. Protein concentrations were determined with Bio-Rad dye reagent using bovine serum albumin (BSA) as the standard. The yields of tag-free Ifn1 at this stage of purification (per liter of culture) were as follows: WT, 4.6 mg; D11A, 17 mg; R50A, 3.5 mg; Y54A, 6 mg; D80A, 10 mg; D174A, 12 mg; and R50N, 8.4 mg.

### Gel filtration

Aliquots of the Ifn1 protein preparations were concentrated by centrifugal ultrafiltration (Amicon Ultra-4; 10 kDa cutoff) to 0.5 mL volume and then gel-filtered through a 24 mL 16/60 HiLoad Superdex 200 Increase 10/300 column equilibrated in buffer D (20 mM Tris-HCl, pH 8.0, 250 mM NaCl, 1 mM DTT, and 10% glycerol) at a flow rate of 0.5 mL/min while collecting 0.5 mL fractions. The peak fractions were stored at −80°C.

### Assay of 5′-nucleotidase activity

Reaction mixtures (50 µL) containing 50 mM Tris-HCl buffer, MgCl_2_, BSA, NMP substrate, and Ifn1 (at concentrations specified in the figure legends) were incubated at 37°C. The reactions were quenched at the times specified by the addition of 1 mL of Malachite Green reagent (BIOMOL Green, from Enzo Biochem), and the absorbance at 620 nm was measured after 20-min incubation at room temperature. The yield of released free phosphate was determined by interpolation of the *A*_620_ values to a phosphate standard curve.

### Inducible overexpression of Ifn1 in fission yeast

We constructed fission yeast multicopy *LEU2* plasmids pREP3X-Ifn1, pREP3X-Ifn1-D11A, pREP41X-Ifn1, and pREP41X-Ifn1-D11A, in which the wild-type *ifn1* ORF or the *D11A* ORF is under the control of the thiamine-repressible high-strength *3x-nmt1* promoter or the medium-strength *41x-nmt1* promoter (20). Fission yeast cells bearing the pREP-Ifn1 plasmids or empty pREP vectors were grown to the mid-log phase in liquid ePMGT(–leu) medium, harvested by centrifugation, washed with water, and resuspended in water at an equivalent *A*_600_. Serial fivefold dilutions were spotted on thiamine-containing ePMGT(–leu) agar medium or thiamine-lacking ePMG(–leu) agar medium. Growth was assessed after incubation for 4 days at 30°C.
